# EphA4 Receptor Tyrosine Kinase Is a Modulator of Onset and Disease Severity of Experimental Autoimmune Encephalomyelitis (EAE)

**DOI:** 10.1371/journal.pone.0055948

**Published:** 2013-02-04

**Authors:** Kathryn M. Munro, Kirsty J. Dixon, Melissa M. Gresle, Anna Jonas, Dennis Kemper, William Doherty, Louis J. Fabri, Catherine M. Owczarek, Martin Pearse, Andrew W. Boyd, Trevor J. Kilpatrick, Helmut Butzkueven, Ann M. Turnley

**Affiliations:** 1 Centre for Neuroscience Research, The University of Melbourne, Victoria, Australia; 2 Department of Anatomy and Neuroscience, The University of Melbourne, Victoria, Australia; 3 Department of Medicine, Melbourne Brain Centre at the Royal Melbourne Hospital, The University of Melbourne, Victoria, Australia; 4 MS Division, Florey Neuroscience Institutes, Melbourne, Victoria, Australia; 5 CSL Ltd, Parkville, Victoria, Australia; 6 Queensland Institute of Medical Research, Brisbane, Queensland; University of Utah School of Medicine, United States of America

## Abstract

The EphA4 receptor tyrosine kinase is a major regulator of axonal growth and astrocyte reactivity and is a possible inflammatory mediator. Given that multiple sclerosis (MS) is primarily an inflammatory demyelinating disease and in mouse models of MS, such as experimental autoimmune encephalomyelitis (EAE), axonal degeneration and reactive gliosis are prominent clinical features, we hypothesised that endogenous EphA4 could play a role in modulating EAE. EAE was induced in EphA4 knockout and wildtype mice using MOG peptide immunisation and clinical severity and histological features of the disease were then compared in lumbar spinal cord sections. EphA4 knockout mice exhibited a markedly less severe clinical course than wildtype mice, with a lower maximum disease grade and a slightly later onset of clinical symptoms. Numbers of infiltrating T cells and macrophages, the number and size of the lesions, and the extent of astrocytic gliosis were similar in both genotypes; however, EphA4 knockout mice appeared to have decreased axonal pathology. Blocking of EphA4 in wildtype mice by administration of soluble EphA4 (EphA4-Fc) as a decoy receptor following induction of EAE produced a delay in onset of clinical symptoms; however, most mice had clinical symptoms of similar severity by 22 days, indicating that EphA4 blocking treatment slowed early EAE disease evolution. Again there were no apparent differences in histopathology. To determine whether the role of EphA4 in modulating EAE was CNS mediated or due to an altered immune response, MOG primed T cells from wildtype and EphA4 knockout mice were passively transferred into naive recipient mice and both were shown to induce disease of equivalent severity. These results are consistent with a non-inflammatory, CNS specific, deleterious effect of EphA4 during neuroinflammation that results in axonal pathology.

## Introduction

Multiple Sclerosis (MS) is an autoimmune, neurodegenerative disease with a complex aetiology. The pathophysiology of MS includes blood brain barrier breakdown, infiltration of T cells, destruction of myelin by macrophages [1], [2], oligodendrocyte apoptosis and astrocytic gliosis [3], [4]. Permanent neurologic disability associated with MS is likely to be caused by axonal injury [5]. The experimental autoimmune encephalomyelitis (EAE) model has been extensively used to examine particular aspects of MS, sharing numerous features such as the presence of multiple inflammatory CNS lesions, axonal damage and astrocytic gliosis [6]. EAE is induced either by inoculation with CNS myelin antigens, such as myelin oligodendrocyte glycoprotein (MOG) (active EAE) or passive transfer of myelin antigen-specific T cells (passive EAE), and the pathological features and clinical disease symptoms of EAE vary according to the species, strain and antigen used [7].

Given that MS and some models of EAE involve axonal injury, we were interested in determining whether factors that regulate axon outgrowth or regeneration may play a role in CNS neuroinflammatory disease. The EphA4 receptor tyrosine kinase is a promising candidate in this regard, as EphA4 knockout mice display extensive axonal regeneration and functional recovery following spinal cord injury [8], an effect also seen when the EphA4 receptor is blocked by administration of soluble EphA4 decoy receptor or the ephrin-A5 ligand [9]. Further, Ephs and ephrins have been localised to macrophages, reactive astrocytes and axons in and around MS lesions [10] and EphA4 is involved in thymus development [11] and is expressed under some conditions in CD4+ and CD8+ T cells [12], [13]. In addition, microarray analysis of the injured spinal cords of EphA4 knockout mice demonstrated that the expression of a number of inflammation-related genes were altered and a lower proportion of Arginase-1 (ARG1)-expressing macrophages were found at the injury site of EphA4 knockout spinal cords *in vivo* [14]. Therefore, EphA4 could also play a role in propagating inflammation and/or axonal damage in neuroinflammatory disease.

The MOG model of EAE in particular is characterised by paralysis, axonal damage and inflammatory lesions in the spinal cord and optic nerves. These lesions contain macrophages / activated microglia, CD4^+^ and CD8^+^ T cells and degenerating and demyelinated axons [15]. Reactive astrocytes are present in and around CNS lesions in MS patients and EAE animals, and similar to the astrocytic response following traumatic CNS injury, the astrocytic response could have positive and negative effects on disease pathology [16]. Axonal loss in the MOG-EAE model begins as early as 7 days post-immunisation, before clinical symptoms are detected, at a time when the extent of inflammatory infiltration is low [17], and axonal injury is not necessarily associated with demyelination [17], [18]. Axon loss occurs not only in spinal cord white matter lesion areas, but also in perilesional areas and normal appearing white matter (NAWM) [19]. Neural damage in all these areas closely correlates with clinical disease severity [20].

Given that Eph-ephrin interactions and the expression of EphA4 specifically, may contribute to the pathology of MS and EAE in a number of ways, including effects in the immune system, a glial cell response and axonal damage, in this manuscript we have assessed the role of EphA4 in regulating EAE severity and development by use of EphA4 knockout animals and blocking of EphA4 using a decoy receptor.

## Materials and Methods

### Ethics statement

Experiments were approved by the University of Melbourne Animal Ethics Committee (approval number 0703922) in accordance with the Australian Code of Practice for the Care and Use of Animals for Scientific Purposes.

### Mice and EAE induction

For analysis of EAE in EphA4 knockout mice, male and female EphA4 knockout mice and their wild-type littermates (C57Bl/6 background) were used. For analysis of the effects of blocking EphA4 or passive transfer of wildtype and EphA4 knockout T cells, C57Bl/6 mice were purchased from Animal Resource Services (Western Australia).

At 8–16 weeks of age mice received a subcutaneous injection of 125 µg MOG_35–55_ peptide (Mimotopes, Australia) emulsified 1:1 (vol/vol) in Complete Freund’s Adjuvant containing 4mg/ml Mycobacterium tuberculosis H37Ra (Difco, Detroit, MI), to both flanks and the base of the tail. Pertussis toxin (300ng in PBS; List Biological, USA) was injected intraperitoneally at the time of induction and a second dose was administered three days later.

EphA4 knockout and wild-type cohorts were age and sex matched. EphA4 mice have an altered gait, exhibiting hopping, rather than reciprocal movement of the hindlimbs. They do not show any signs of paralysis or limb weakness, the signs used for scoring of EAE severity. Animals were weighed, monitored and clinically assessed according to the following grading scale: 0 = no symptoms; 1 = distal tail weakness; 1.5 = tail weakness and some hindlimb weakness; 2 = complete tail paralysis; 2.5 = complete tail paralysis and partial hindlimb weakness; 3 = complete hindlimb weakness; 3.5 = inability to right when placed on back or significant forelimb weakness; 4 = euthanize or spontaneous death [20], [21]. Mice were euthanized if they lost 20% of their starting weight, displayed a clinical score of 3 for 72 hours or reached a clinical score of 3.5. Mice were examined for up to 23 days post-immunisation. Numbers of mice used: EphA4 knockout *n* = 16; wild-type *n* = 18; C57Bl/6 treated with EphA4-Fc blocker *n* = 26, C57Bl/6 treated with control IgG *n* = 27; passive transfer of T cells: EphA4 knockout donors *n* = 5, wildtype donors *n* = 7, C57Bl/6 recipients *n* = 15.

### Administration of EphA4 blocker

A soluble EphA4 receptor decoy, EphA4-Fc, was used to block EphA4 interactions. The extracellular domain of mouse EphA4 (amino acids 1-546 of NP_031962.2) was fused to mouse immunoglobulin Fc (supplied by CSL Ltd, Victoria, Australia), as previously described [9]. From the day of EAE induction, 500 µg EphA4-Fc blocker or mouse IgG control (Sigma-Aldrich, Australia) was administered daily by intraperitoneal (IP) injection, until 22 days after disease induction or death of the mouse.

### EAE induction by T cell passive transfer

At 10 days following the MOG EAE induction in EphA4 knockout and wildtype mice, mice were killed by IP injection of Lethabarb (sodium pentobarbitone, 325mg/ml). The draining lymph nodes and spleen were then removed, ground and washed on to a 70 µM nylon sieve, and then centrifuged at 1500rpm for 5min. Red blood cells were lysed by re-suspending the cell pellet in ammonium chloride potassium carbonate lysis buffer (0.15M NH4Cl, 10mM KHCO3, 2mM Na4EDTA dH_2_0), 1ml per donor mouse, and incubating on ice for 5 min. The reaction was stopped by adding 9ml complete media and mixing. The debris was left to settle to the bottom of the tube for 2min, and cell suspension was transferred into a new tube and centrifuged at 1500rpm for 5 min. The cell pellet was washed a further two times in PBS (Gibco) containing 0.5% FBS (Gibco), and then re-suspended in complete DMEM containing 50 µg of MOG35-55 peptide and 20ng of IL-2, to obtain 6x10^6^ cells per ml for plating. The cells were then left to incubate for 48h at 37^o^C in a 5% CO2 incubator. After this time, the non-adherent T-lymphocyte cells were collected and injected I.P. into wild type donor mice at a concentration of 2x10^6^ cells in 0.1ml PBS per mouse. On the same day, the mice were injected I.P. with 300ng pertussis toxin on the side opposite to the site of T-lymphocyte cell injection. A second dose of pertussis toxin was administered 2 days later. Assessments of disease severity were performed daily as described above.

### Histology

Mice received a lethal injection of sodium pentobarbital (Lethabarb, 320 mg/kg I.P.) and were transcardially perfused with 0.1M PBS and 4% paraformaldehyde (PFA, Sigma-Aldrich) in PBS. Spinal cord tissue was post-fixed in 4% PFA for two hours, placed in 20% sucrose in PBS overnight, then frozen in Tissue-Tek O.C.T. compound (Sakura Finetek, USA). Transverse sections (10 µm) of spinal cords were taken from the lumbar enlargement and collected onto Superfrost Plus slides (Lomb Scientific, Aust.) and stored at −80°C until used. Every fifth slide was stained with haematoxylin and eosin to assess tissue morphology and the presence of white matter lesions.

### Immunohistochemistry

#### Fluorescent immunohistochemistry

Cryosections were thawed at room temperature, rinsed in PBS, and incubated for a minimum of thirty minutes in blocking solution. For the majority of experiments, the blocking solution used was PBS containing 2% normal goat serum (NGS, Invitrogen, Aust.), 2% foetal calf serum (FCS, Thermo Scientific, Aust.) and 0.2% Triton X100 (Ajax Finechem, Aust.). For ARG1 and CD11b double-labelling, the blocking solution used was PBS containing 2% bovine serum albumin (BSA, Sigma-Aldrich) and 0.2% Tween-20. Sections were incubated with primary antibody diluted in blocking solution overnight at room temperature; primary antibodies and their dilutions were as follows: rat anti-CD11b (1:500; Millipore), goat anti-ARG1 (1∶250; Santa Cruz), rabbit anti-CD3 (1∶500; Abcam), rabbit anti-GFAP (1∶500, DAKO), mouse anti-GFAP (1∶500, Millipore), rabbit anti-EphA4 (1∶300; F88 antiserum, raised against a peptide corresponding to amino acids 938–953 of the intracellular SAM domain of EphA4-GenBank accession number NM007936; [8]).

Sections were washed in PBS three times for a minimum of five minutes, then incubated with a fluorophore-tagged secondary antibody diluted in PBS for one hour at room temperature. For ARG1 and CD11b double-labelling, sections were incubated with the secondary antibody for ARG1 (donkey anti-goat Cy3) before being incubated with the secondary antibody for CD11b (goat anti-rat Alexa Flour 488), to avoid cross-reactivity. Secondary antibodies and their dilutions: goat anti-rat IgG-AlexaFluor 488 (1∶500; Invitrogen) donkey anti-rabbit IgG-AlexaFluor 594 (1∶500; Invitrogen). Cell nuclei were counterstained with DAPI (Sigma). Secondary antibody specificity was determined by omitting the primary antibody. Fluorescent immunohistochemistry was imaged with a Zeiss Meta confocal microscope mounted on a Zeiss Axioplan upright microscope.

#### Diaminobenzadine (DAB) labelled immunohistochemistry

Cryosections were thawed, washed in PBS and incubated in 0.3% H_2_O_2_ in PBS for twenty minutes to block endogenous peroxidase activity. Sections were then incubated for a minimum of thirty minutes in a blocking solution of PBS containing 2% NGS, 2% FCS and 0.2% Triton X100. Sections were incubated with rabbit anti-GFAP antibody diluted in blocking solution overnight at room temperature. Sections were washed in PBS three times for a minimum of five minutes and incubated with biotinylated goat anti-rabbit IgG (1:500; Vector Laboratories, USA) diluted in PBS for one hour at room temperature. Sections were rinsed in PBS three times then incubated with an avidin-biotin solution (Vectastain Elite ABC Kit, Vector, USA) for one hour at room temperature. Sections were incubated with DAB (Liquid DAB Substrate Chromogen System, Dako, USA) for 10 minutes to visualise the labelling then rinsed in H_2_O. Secondary antibody specificity was determined by omitting the primary antibody. Immunohistochemical staining was imaged using bright field microscopy.

### Histological analysis

#### T cell quantitation

CD11b immunostaining for macrophages / activated microglia was used to identify areas of white matter inflammatory infiltration. The density of CD3^+^ T lymphocytes was analysed in lesion and perilesional areas. Images were taken on a confocal microscope at x40 magnification (a field of 210 µm^2^), and coded for blinded analysis. Between 6 and 15 fields were analysed per mouse and the density of CD3^+^ cells within a field was converted to cells/100 µm^2^. CD3^+^ cells were counted when they co-localised with a DAPI nuclear counterstain.

#### Analysis of CD11b+ area

CD11b+ white matter lesions were quantified as a proportion of spinal cord area. A minimum of three sections were analysed per mouse. Tiled images of whole spinal cord sections taken on a confocal microscope at x40 magnification were compiled and coded for blinded analysis. CD11b+ lesion areas in the white matter were traced using LSM software (Zeiss LSM Image Browser Version 4.0.0.241) and quantified as a proportion of whole spinal cord section area.

#### CD11b density counts

The density of CD11b+ cells was analysed within white matter lesion areas. Images were taken on a confocal microscope at x40 magnification, and coded for blinded analysis. Twelve fields were analysed per mouse; in mice with low or no clear inflammatory infiltrate, cell counts were conducted in equivalent areas of white matter.

The density of CD11b+ cells within a field was converted to cells/100 µm^2^. CD11b+ cells were counted when they co-localised with a DAPI nuclear counterstain.

#### ARG1+ macrophage / activated microglia counts

The proportion of ARG1 immunoreactive cells which co-localised with CD11b+ macrophages / activated microglia was counted within white matter inflammatory lesion areas. Images were taken on a confocal microscope at x40 magnification, and coded for blinded analysis. Twelve fields were analysed per mouse; only mice with robust inflammatory lesions were included. CD11b+ cells with a DAPI nuclear counterstain were counted as either ARG1-positive or ARG1-negative.

#### Analysis of astrocytic gliosis

GFAP immunoreactivity was visualised with DAB. Images were taken at x100 magnification in lesion areas and in normal appearing white matter. Images were coded for blinded analysis and the area of positive staining was determined using the thresholding function in ImageJ (Rasband, W.S., ImageJ, U.S. National Institutes of Health, Bethesda, Maryland, USA, http://imagej.nih.gov/ij, 1997–2011).

#### Quantification of axonal area and number

For axonal counts in dorsal columns of spinal cords, Karnovsky-fixed toluidine-blue stained nerve cross-section were photographed at 100 times magnification on a Zeiss Axioplan 2 Imaging system (Axiovision Software Release 4.4; Carl Zeiss Pty Ltd, Oberkochen, DE). Images were imported into ImageJ for cropping. Images were analysed in MetaMorph v7.7.5 (Molecular Devices, USA). The image was initially smoothened using a median filter (5,5,1). Axons were thresholded based on intensity and a binary image was created. The resulting binary was further filtered to remove artefacts (Open-Close [circle 6 pixels]) and any holes were filled using a holes filter. The resulting image was then filtered to select axons (shape factor >0.4), which were finally filtered to select only axons that were associated with a myelin sheath. The number of such axons per unit area was then quantitated and their area measured using Image J.

### pNF-H ELISA

The blood samples collected at the time of culling were used in the pNF-H ELISA, as previously described [20]

### Statistics

Statistics were generated using 2-Way ANOVA with Bonferroni Post-hoc test or t-test in GraphPad Prism (version 4.03, GraphPad Software Inc., USA) and values are represented as mean±standard error of the mean (SEM). The coefficients of determination (r^2^) were calculated to evaluate how well the highest clinical score correlated with histological pathological features. Results were considered statistically significant at *p*<0.05.

## Results

To examine the role of EphA4 in modulating EAE, 3 different experimental paradigms were used: comparison of EphA4 knockout versus wildtype mice, blocking of EphA4 by the use of a soluble decoy receptor (EphA4-Fc) and passive transfer of T cells from EphA4 knockout and wildtype mice to determine whether knockout T-cells could be sufficiently primed to passively transfer the disease.

### EphA4 is expressed on astrocytes at EAE lesions

**Figure 1:**
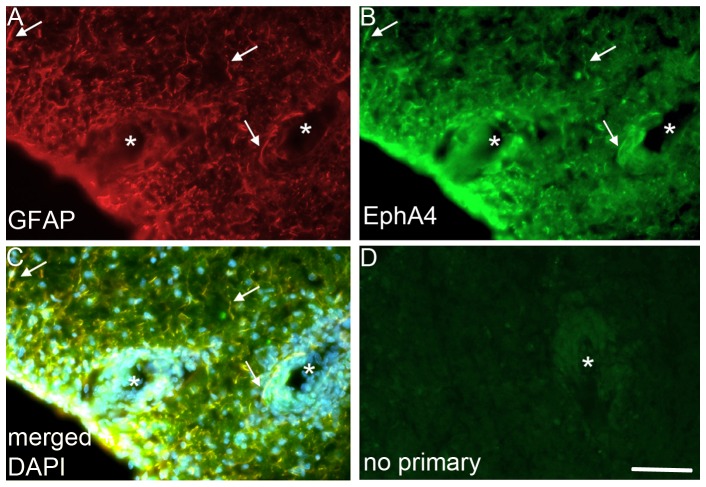
EphA4 is expressed on GFAP positive astrocytes surrounding EAE lesions. Spinal cords from C57BL/6 mice with MOG-induced EAE were immunostained for GFAP and EphA4 expression. a) GFAP expressing astrocytes from these mice also expressed b) EphA4. c) merged b & c, plus DAPI to show nuclei. d) No overt background was present in the absence of primary EphA4 antibody. Scale bar; 100 µm. Lesions are indicated by asterisks. Arrows indicate examples of EphA4-expressing astrocytes.

In order to assess whether EphA4 was expressed on astrocytes in EAE lesions, spinal cord sections from wildtype mice were immunostained for EphA4 expression. EphA4 was expressed on GFAP positive astrocytes surrounding EAE lesions (Fig. 1).

### EphA4 knockout mice have an attenuated response to EAE

**Figure 2:**
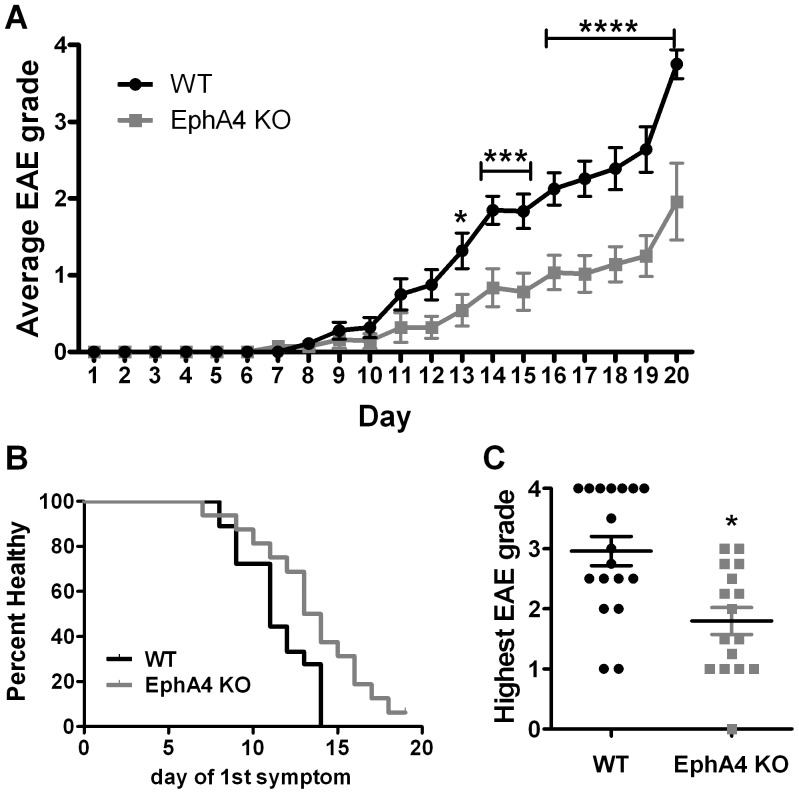
Clinical scoring of EAE-affected wild-type and EphA4 knockout mice. Paralysis in EAE-affected mice was assessed by clinical scoring up to 20 days post-immunisation. **A)** EphA4 knockout (KO) mice had a lower average daily clinical score than wild-type (WT) mice between 12 and 20 days post-immunisation (2-Way Repeated Measures ANOVA with Bonferroni post-hoc analysis, *p<0.05, ***p<0.001, ****p<0.0001). **B)** EphA4 knockout mice had a significantly delay in onset of symptoms; *p* = 0.016, Log-rank (Mantel-Cox) test. **C)** The highest clinical score reached within 20 days post-immunisation was on average significantly lower in EphA4 knockout compared to wild-type mice (unpaired t-test, *p<0.05). Results show mean±SEM of *n* = 18 wild-type and *n* = 16 EphA4 knockout mice.

Wild-type (*n* = 18) and EphA4 knockout (*n* = 16) mice were assessed for symptoms of paralysis for up to 20 days following EAE immunisation. Experiments were conducted using two cohorts of mice, each with similar numbers of wild-type and EphA4 knockout mice, and the data was combined. On average, wild-type mice first displayed neurological symptoms on day 11.4 and EphA4 knockout mice on day 13.2. Between 13 and 20 days post-immunisation, the mean clinical grade was significantly and substantially lower in the EphA4 knockout compared to the wild-type group (Table 1; Fig. 2a; 2-way Repeated Measures ANOVA effect of genotype *p*<0.001) and the day of appearance of the first symptom was delayed (Table 1; Fig. 2b). The mean highest score reached by EAE-affected wild-type mice was significantly higher (*p* = 0.0015) than that reached by EphA4 knockout mice (Table 1; Fig. 2c; mean scores of 3 and 1.8 respectively), with 7 out of 18 wild-type mice reaching a grade of 4 and no EphA4 knockout mice reaching this grade. The time point post-immunisation when the highest mean clinical score occurred was day 16.5 in wild-type and day 15.9 in EphA4 knockout mice.

**Table 1:** EAE grading data for wildtype (WT) and EphA4 knockout (KO) mice.

Genotype	Day of onset	Peak disease grade	Day of peak disease
*Wild-type mean±sem*	*11.39±0.51*	*2.96±0.24*	*16.50±0.80*
*EphA4 KO mean±sem*	*13.20±0.72*	1.79±0.21*	*15.93±0.82*

WT *n* = 18 (11 female, 7 male); KO *n* = 16 (10 female, 6 male)

### No difference in T lymphocyte infiltration in EAE-affected wild-type and EphA4 knockout spinal cords at 20 days post-immunisation

As spinal cords were not collected from mice with a clinical score of 4 (found dead or required immediate killing), the histological analysis of inflammation and gliosis presented below does not encompass the most severely affected wild-type samples. Therefore the histological comparisons of EAE-affected wild-type and EphA4 knockout mice were conducted in animals with similar disease grades.

**Figure 3:**
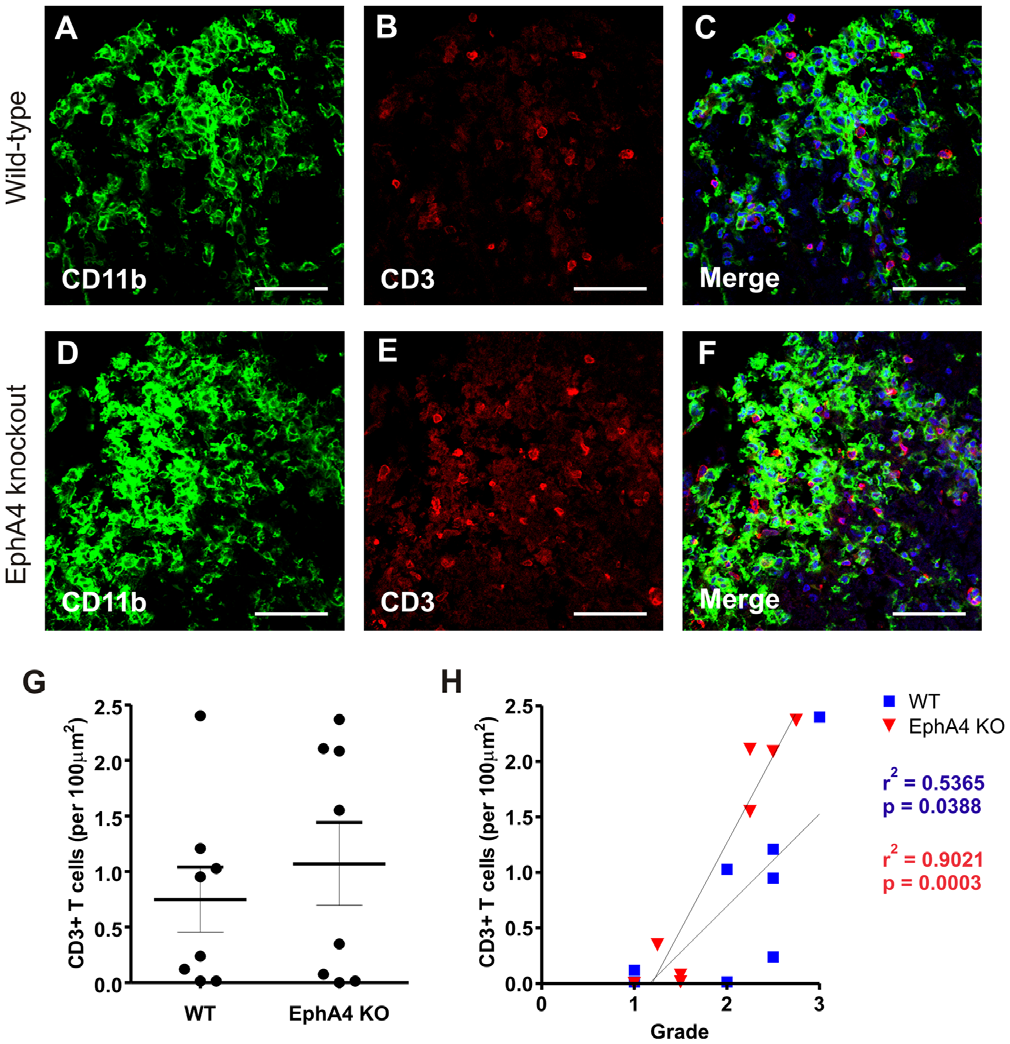
T lymphocyte infiltration in EAE-affected wild-type and EphA4 knockout spinal cords at 20 days post-immunisation. Inflammatory white matter lesions in both EAE-affected wild-type and EphA4 knockout lumbar spinal cords (visualised as CD11b+ areas in **A**, **D**) display a robust infiltration of CD3^+^ T cells (**B**, **E**, and merged in **D**, **F**). There was no significant difference (*p*>0.05) in the number of T cells present in the spinal cord lesions of wild-type and EphA4 knockout mice (**G**). In both genotypes, there was a significant (*p*<0.05) correlation between the number of T cells present in lesions and the highest clinical grade reached per mouse (**H**). Merged images include DAPI nuclear counterstain. Scale bars A−F = 50 µm. Results in G show the mean±SEM of *n* = 8 wild-type (WT) and *n* = 8 EphA4 knockout (KO) spinal cords of mice of clinical grades 1 to 3.

To identify inflammatory white matter lesions in EAE-affected mice, sections were immunostained for CD11b to label macrophages / activated microglia. Double-labelling with the pan T lymphocyte marker CD3 identified robust T cell infiltration within and immediately adjacent to CD11b+ lesions in both genotypes (Fig. 3a–f). There was no significant difference (*p*>0.05) between genotypes in the average number of T cells per 100 µm^2^ in lesion and peri-lesion areas (Fig. 3g). In both EAE-affected wild-type and EphA4 knockout groups, there was a significant correlation (wild-type, *p* = 0.0388; EphA4 knockout, *p* = 0.0003) between the amount of T cell infiltration and the highest clinical grade reached per mouse (Fig. 3h).

### No difference in area and density of infiltrating macrophages / activated microglia in EAE-affected wild-type and EphA4 knockout spinal cords

**Figure 4:**
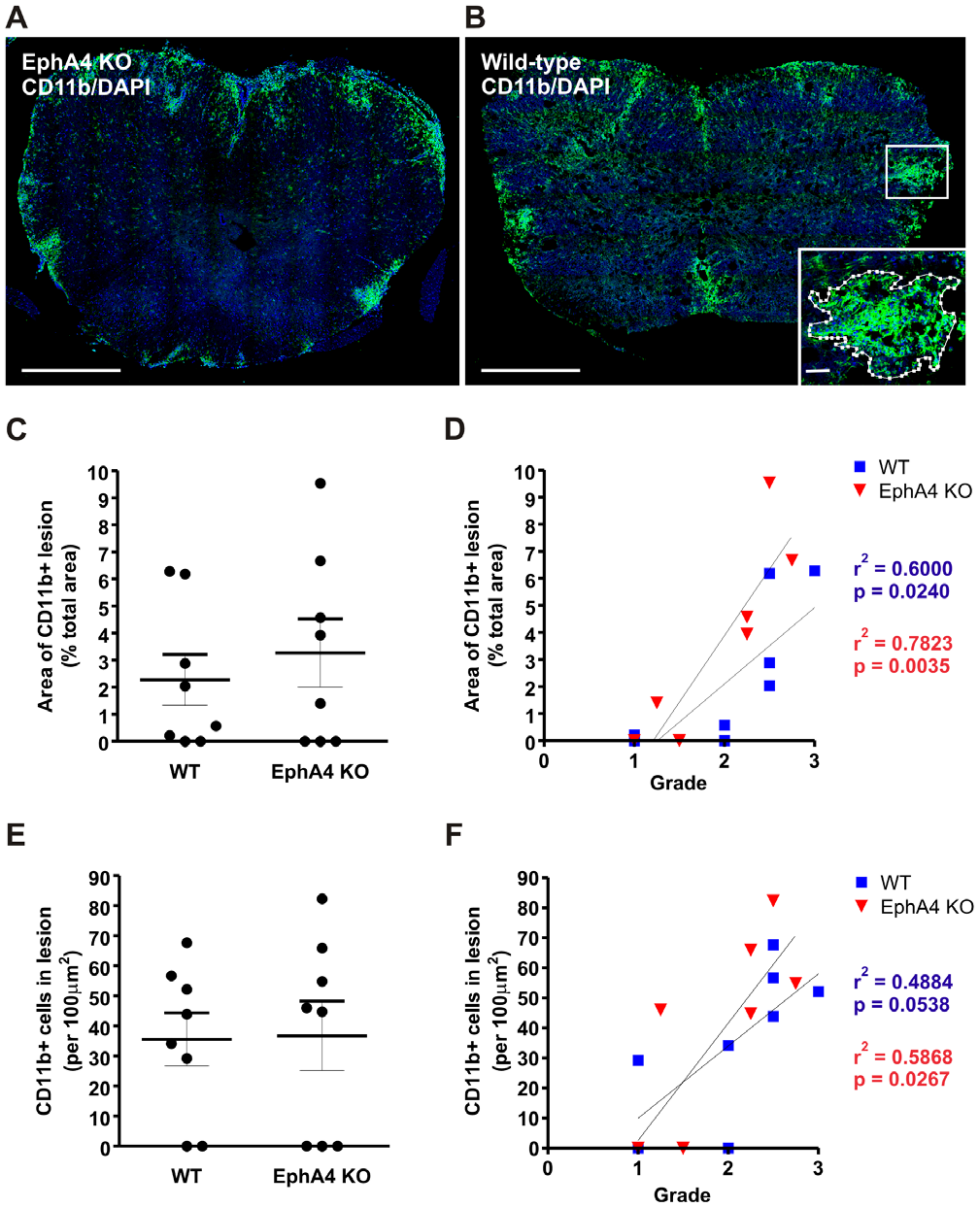
Area and density of infiltrating macrophages / activated microglia in EAE-affected wild-type and EphA4 knockout spinal cords at 20 days post-immunisation. Gross areas of inflammatory infiltrate were determined in whole sections of EphA4 knockout (KO, A) and wild-type (B) lumbar spinal cords by tracing around CD11b+ lesion areas at x40 magnification (inset in B). CD11b immunostaining is shown in green with a DAPI nuclear counterstain. There was no significant difference (*p*>0.05) between wild-type (WT) and EphA4 knockout mice in the average area of CD11b+ lesions as a proportion of lumbar spinal cord area (C). In both EAE-affected genotypes, there was a significant (*p*<0.05) correlation between the proportional area of CD11b+ lesions and the highest clinical grade reached per mouse (D). There was no difference between genotypes in the density of CD11b+ cells within lesion areas (E). There was a significant correlation between the density of CD11b+ cells and the highest clinical grade reached in EphA4 knockout mice, and a similar trend was seen in wild-type mice (F). Scale bars in A and B = 500 µm, inset in B = 50 µm. Results in C and E show the mean±SEM of *n* = 8 wild-type and *n* = 8 EphA4 knockout spinal cords of mice of clinical grades 1 to 3.

The total area of inflammatory infiltrate was estimated by examining the area of staining for macrophages / activated microglia, the most numerous immune cell types present in MOG-EAE white matter lesions. Areas of dense CD11b immunoreactivity were traced at x40 magnification and quantified as a proportion of the total spinal cord section area (Fig. 4a,b). There was no significant difference (*p*>0.05) between EAE-affected wild-type and EphA4 knockout mice in the mean area of CD11b+ lesions as a proportion of spinal cord area (Fig. 4c). In both genotypes, there was a significant correlation (wild-type, *p* = 0.024; EphA4 knockout, *p* = 0.004) between the proportional area of CD11b+ lesions and the highest clinical score reached by a given mouse (Fig. 4d). Within CD11b+ white matter lesions, the density of macrophages / activated microglia was quantified. There was no difference between genotypes in the density of CD11b+ cells within lesion areas (Fig. 4e). There was a significant correlation between the density of CD11b+ cells and the highest clinical grade reached in EphA4 knockout mice (*p* = 0.03) and in wild-type mice (p = 0.05) (Fig. 4f). Spinal cord sections were also double labelled with CD11b and the subtype marker ARG1, which we found to be differentially expressed in wildtype and EphA4 knockout spinal cord following spinal cord injury [14] and which has also been shown to modulate EAE clinical severity [22]. A subset of CD11b+ cells displayed ARG1 immunoreactivity in EAE lesions in both genotypes but there were no significant differences (*p*>0.05) (data not shown).

### No difference in astrocytic gliosis in EAE-affected wild-type and EphA4 knockout spinal cords

**Figure 5:**
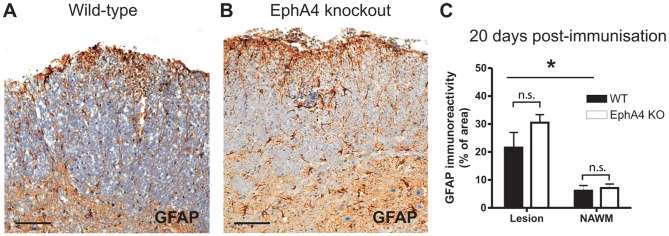
Astrocytic gliosis in EAE-affected wild-type and EphA4 knockout spinal cords. Astrocytic gliosis is present in lesion areas in EAE-affected wild-type (**A**) and EphA4 knockout (**B**) spinal cords at 20 days post-immunisation, as evidenced by increased GFAP expression. There was a significantly greater GFAP+ area in white matter lesion areas compared to NAWM in both genotypes (*p<0.05; wild-type (WT), EphA4 knockout (KO) but there was no significant difference (n.s.) between genotypes (**C**). Results in C show the mean±SEM of *n* = 6 wild-type and *n* = 5 EphA4 knockout spinal cords of mice of clinical grades 1 to 3 for lesion measurements, and *n* = 8 wild-type and *n* = 8 EphA4 knockout spinal cords of mice of clinical grades 1 to 3 for NAWM measurements. Scale bars: A, B  =  100 µm.

At 20 days post-immunisation, astrocytic gliosis was present in inflammatory white matter lesion areas in EAE-affected wild-type and EphA4 knockout lumbar spinal cords, as demonstrated by increased GFAP immunoreactivity (Fig. 5a,b). The proportional area of GFAP immunoreactivity was significantly increased in lesions compared to NAWM in both wild-type and EphA4 knockout tissue (wild-type lesion vs. wild-type NAWM, *p* = 0.0105; EphA4 knockout lesion vs. EphA4 knockout NAWM, *p* = 0.0001), however there was no significant difference (*p*>0.05) between genotypes (Fig. 5c).

### Axonal damage in EAE-affected wild-type and EphA4 knockout spinal cords

**Figure 6:**
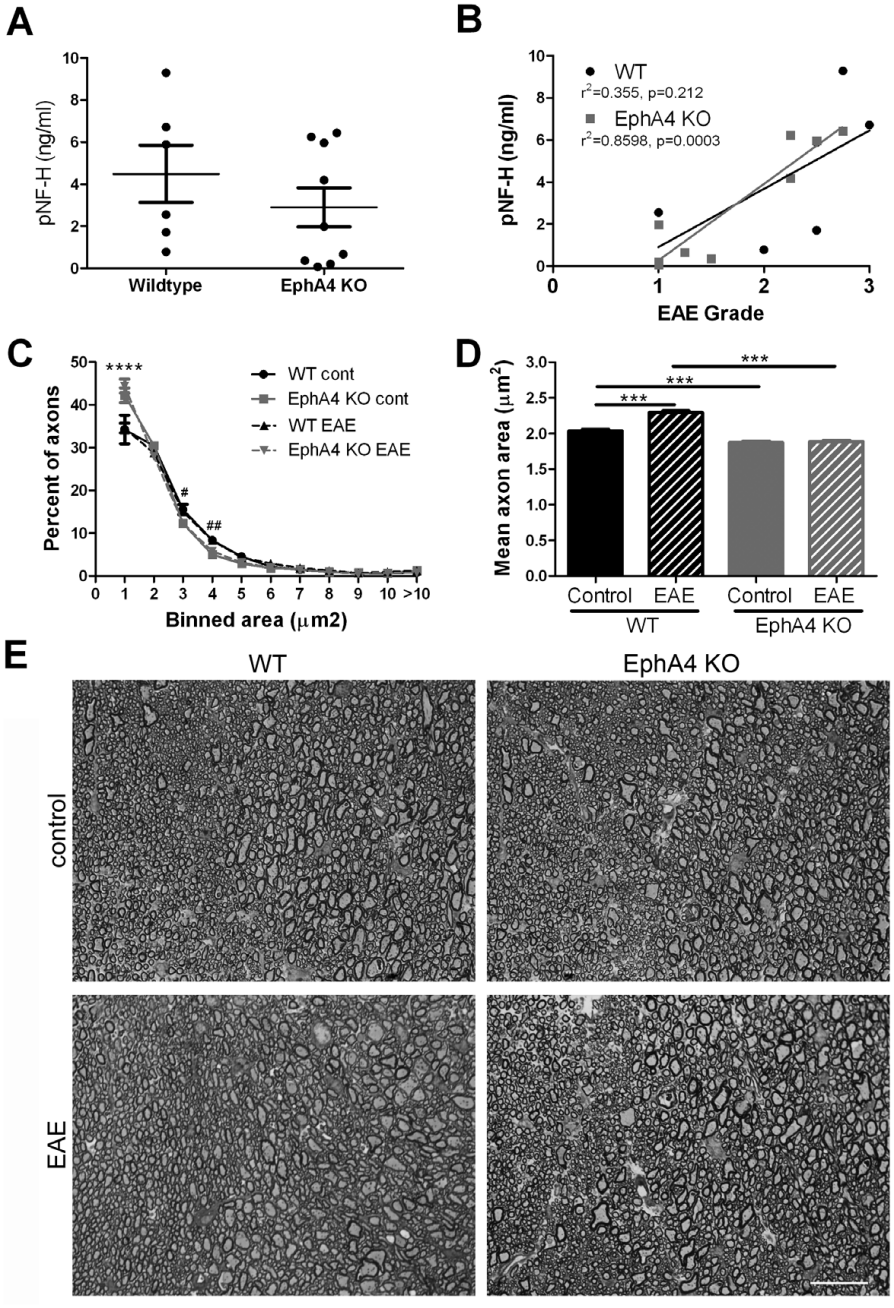
Axonal damage in EAE-affected wild-type and EphA4 knockout spinal cords. (**A,B**) Blood samples from EAE-affected wild-type (WT, *n* = 6) and EphA4 knockout (KO, *n* = 9) mice were taken at 20 days post-immunisation and pNF-H levels were measured as an indicator of axonal injury. **A**) There was no significant difference in mean pNF-H levels between genotypes. **B**) In EphA4 knockout mice there was a significant correlation between pNF-H levels and highest clinical score reached per mouse; a similar non-significant trend was observed in wild-type mice. **C,D)** Analysis of axonal diameter in the dorsal funiculus of control and EAE-affected mice indicated that KO mice had a greater number of small diameter axons than wildtype control and EAE-affected WT mice. **C)** The distribution of axon diameters did not differ between control and EAE-affected mice but WT mice had fewer axons in the smallest category in either case. 2-way ANOVA p<0.0001, ****p<0.0001 and ##p<0.01, #p<0.05 for EAE-affected mice only. **D)** The mean diameter of WT but not KO axons increased with EAE compared to control mice, indicative of increased axonal pathology in the wildtype mice and a relative sparing of KO axons. 1-way ANOVA p<0.0001, ***p<0.001. **E)** Representative images of axons in the dorsal funiculus in normal control and EAE-affected WT and EphA4 KO mice. Scale bar 20 µm.

pNF-H levels in serum samples taken from EAE-affected wild-type and EphA4 knockout mice at 20 days post-immunisation were measured as an indicator of axonal injury. There was a trend to decreased pNF-H levels in EphA4 knockout mice (44% of EphA4 knockout mice had levels of <1ng/ml, while 16% of wildtypes had similar low levels), however the mean difference in pNF-H levels between genotypes was not significant (Fig. 6a). A significant correlation was observed between the level of p-NF-H in the serum and the highest clinical score reached per mouse in the EphA4 knockout group (*p* = 0.0003); wild-type samples followed a similar trend but did not reach significance (*p* > 0.05) (Fig. 6b). Analysis of the distribution of axonal area in control and EAE-affected mice indicated that EphA4 knockout mice had an increased proportion of small axons relative to wildtype mice (Fig. 6c). The mean axon area increased in EAE-affected wildtype mice but not EphA4 knockout mice, compared to their respective non-diseased control mice, probably indicating EAE-related axonal swelling and hypertrophy in wildtype mice which was not apparent in EphA4 knockout axons (Fig. 6d,e). The median diameter of EAE-affected wildtype axons (*n* = 5 mice) was 1.48+/−0.06 µm^2^ and EphA4 knockout axons (*n* = 6 mice) was 1.17+/−0.07 µm^2^ (*p = *0.01). In addition, we did not see any gross differences in myelination between wildtype and EphA4 knockout mice (Fig. S1).

### Blocking of EphA4 in wildtype mice delays initial EAE severity

**Figure 7:**
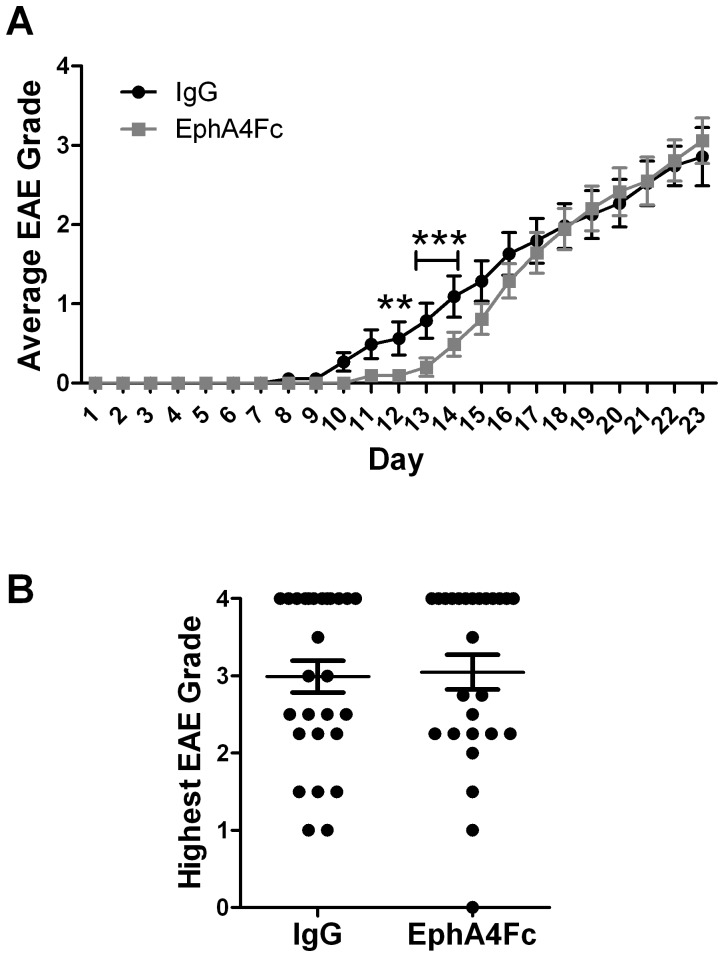
Clinical scoring of EAE-affected IgG control and EphA4Fc treated mice. Paralysis in EAE-affected mice was assessed by clinical scoring up to 23 days post-immunisation. **A)** EphA4Fc treated mice had a delayed onset of clinical symptoms (2-Way Repeated Measures ANOVA with Bonferroni post-hoc test **p<0.01, ***p<0.001). **B)** The highest clinical score reached within 23 days post-immunisation was similar in control and treated mice (unpaired t-test, *p<0.05). Results show mean±SEM of *n* = 27 IgG control and *n* = 26 EphA4Fc treated mice.

As EAE induction in EphA4 knockout mice decreased the severity of clinical symptoms we next assessed whether blocking EphA4 in wildtype mice would also decrease clinical severity. To do this, we administered a decoy receptor, EphA4Fc, daily for up to 22 days to block EphA4 interactions with ephrin ligands, as we previously used following spinal cord injury [9]. This delayed severity of clinical symptoms for 3–4 days but treated animals eventually reached the same EAE grade as IgG treated control mice (Fig. 7 and Table 2). Further, there were no differences detected in inflammatory lesion number or size, astrocytic gliosis or plasma pNF-H levels between EphA4Fc treated or control animals with similar clinical grades (data not shown).

**Table 2:** EAE grading data for control (IgG) and EphA4-Fc treated mice.

Treatment	Day of Onset	Peak disease grade	Day of peak disease
*IgG treated mean±sem*	*15.33±0.90*	*2.99±0.21*	*19.63±0.60*
*EphA4-Fc mean±sem*	*15.35±0.85*	*3.05±0.22*	*20.16±0.53*

IgG control *n* = 27 (11 female, 16 male), EphA4-Fc *n* = 26 (9 female, 17 male)

### Is the role of EphA4 in EAE due to CNS or T cell mediated effects?

Because EphA4 is expressed in the CNS and also plays a role in T cell development [11] and EAE is a T cell-mediated disease, we wished to determine whether EphA4 knockout T cells were able to induce EAE, to indicate whether the effect of EphA4 on EAE severity was due to central or peripheral effects. To do this we used the passive transfer model of EAE, whereby T cells from EAE-affected mice are transferred to naive recipient mice to induce disease. In this case, we used T cells from EAE-affected wildtype and EphA4 knockout mice and compared their ability to induce EAE in wildtype mice. T cells from 7/7 wildtype and 5/5 knockout mice induced mild EAE in wildtype recipient mice, reaching Grade 1–1.5 by 26 days after passive transfer (data not shown). Therefore, EphA4 knockout T cells mice were able to transfer disease to wildtype mice, indicating that they were not impaired in this regard.

## Discussion

EphA4 has been shown to regulate recovery from spinal cord injury (Goldshmit, Fabes, etc refs) and may play a role in ischemia [23]. We have shown here that EphA4 knockout mice have markedly attenuated MOG peptide-induced EAE. The onset of neurological symptoms occurred within a similar timeframe in both wild-type and EphA4 knockout mice; however soon after the onset of symptoms the average daily clinical score was lower in EphA4 knockout compared to wild-type mice. Indeed, none of the EphA4 knockout mice reached the maximal clinical score of 4, unlike a large proportion of their wildtype littermates. However, while administration of an EphA4 blocker, EphA4-Fc, also attenuated the EAE response in wildtype mice, this was only a temporary effect and EphA4-Fc treated mice attained a clinical severity equal to that found in IgG administered control mice within a few days of disease onset, possibly due to incomplete penetration of the fusion protein into the CNS parenchyma. Whether or not EphA4 knockout mice would eventually reach disease of a similar clinical severity as wildtype mice will need to be determined in cohorts of mice with a longer timecourse. However, although the average clinical severity was highest at the final 20 day timepoint, for many individual mice the day the highest clinical grade was reached was around day 16, with no significant difference between wildtype and EphA4 null mice (Table 1).

### Role of EphA4 in EAE severity

While we are not certain of the definitive mechanism by which EphA4 knockout mice showed reduced EAE severity, with an absence of any mice reaching the maximal clinical score of 4 by 20 days post-immunisation, the likely effects of EphA4 occur within the CNS parenchyma, as passive transfer of T cells from either wildtype or EphA4 knockout mice to naive wildtype mice induced comparable levels of EAE. This is consistent with a lack of effect of EphA4 on T cell priming but further in vitro and in vivo studies are required to fully determine whether or not EphA4 has other effects on T cell function, including transfer of T cells to EphA4 null animals to rule out any peripheral defect.

Within the CNS parenchyma, EphA4 may play a role in a number of cellular processes, ranging from effects on inflammatory responses to axonal degeneration and/or regeneration. Comparison of these processes between genotypes was compromised by the absence of samples from wild-type mice with a maximal clinical score of 4 (death); as a result, the most severely affected animals were not available for subsequent analysis and, instead, wild-type and EphA4 knockout mice of comparable grades were analysed. No significant histological differences in lesion area or composition were found between groups, illustrating that loss of EphA4 does not significantly affect the cellular composition of lesions in mice of different genotypes with comparable disease severity. Although there was no significant difference in pNF-H serum levels, there appeared to be relatively less injury of axons in the EAE-affected EphA4 knockout mice. Unlike wildtype mice, which showed an increase in mean axonal area with EAE, the EphA4 knockout mice showed no significant difference between control and EAE axonal areas, indicating that this may be one of the main mechanisms by which lack of EphA4 promotes reduced clinical EAE severity. However, some caution needs to be used in interpreting these results, as the EphA4 knockout mice had, on average, axons of smaller area. The axons were measured in the dorsal funiculus, an area that shows reliable axonal degeneration in EAE and which is responsible for motor movements in mice. However, this region also shows anatomical abnormalities in EphA4 knockout mice, with the corticospinal tract axons showing altered midline crossing and reorganisation of their projections [24], [25]. It is possible that such basally altered axonal characteristics in the EphA4 knockout mice may have an effect on EAE disease severity but our finding that blocking of EphA4 in wildtype animals with decoy receptor also decreased/delayed EAE severity suggests that effects of EphA4 on basal axon size may be independent from effects following EAE induction. Further, we have previously shown that EphA4 regulates neurite outgrowth and axonal regeneration spinal cord injury [8], [9] so an effect on axonal responses is in keeping with these findings. However, we did not find any significant effect of EphA4 on axonal degeneration following spinal cord injury [9] and axonal regeneration is unlikely in the timelines studied in the EAE experiments described herein. Further work is required to fully elucidate the role of axonal damage in the context of EAE following EphA4 modulation.

### Inflammation and EAE – T cells

At 20 days post-immunisation, both wild-type and EphA4 knockout mice displayed robust T lymphocyte infiltration in lesion and peri-lesion areas which positively correlated with clinical grade. Although EphA4 knockout mice are known to have a small thymus and altered T cell development at four weeks of age [11] we demonstrate no effect of T cell priming, T cell migration into the CNS or induction of lesion formation in EAE.

### Inflammation and EAE – macrophages/microglia

Activated microglia and macrophages have been shown to have both negative and positive effects on the pathogenesis of MS and EAE. Microglial activation in MOG-EAE is observed as early as 7 days post-induction, before the onset of symptoms [17], [26] and the number of activated microglia and infiltrating macrophages are highest at the peak of clinical symptoms [19]. Activated microglia and macrophages are primarily thought to have detrimental effects on MS and EAE disease progression, and decreasing microglial activation and macrophage infiltration can lessen the severity of EAE [27], [28]. Macrophages and activated microglia can also have positive effects on the pathogenesis of MS and EAE. Microglia release trophic factors and protective mediators, and may recruit neural precursor cells to sites of damage and promote oligodendrogenesis [29], [30].

### Potential contribution of parenchymal cells to altered EAE outcomes

The attenuated neurological symptoms observed in EAE-affected EphA4 knockout mice may be directly or indirectly due to differential responses of non-inflammatory CNS parenchymal cells, namely neurons, astrocytes or oligodendrocytes. GFAP immunoreactivity in lesion areas was similarly upregulated in EAE-affected wild-type and EphA4 knockout mice at 20 days post-immunisation, indicating no overt alterations in the astrocytic response in EphA4 knockout mice, although expression of EphA4 by these cells would affect axonal interactions. Axonal injury occurs prominently within lesion sites, but as the disease progresses axonal loss and damage is seen in peri-lesional areas and areas of NAWM [19]. The lack of increased area of EphA4 knockout axons in EAE is indicative of protection from axonal damage in these animals. To further explore the relative roles of EphA4 expression in axons versus glia, a conditional knockout approach will need to be pursued.

### Conclusions

The EphA4 receptor regulates disease severity and lack of the receptor delays onset in EphA4 knockout mice. This effect of EphA4 is likely to be CNS based, as there was no obvious difference in inflammatory infiltration and EphA4 knockout T cells were able to passively transfer EAE to naive wildtype animals. This appears to be due to protection from axonal damage, although the mechanisms for this are yet to be determined. Candidates for regulating the effect are astrocytes, which express EphA4 surrounding EAE inflammatory lesions. Although there was no obvious effect on levels of GFAP expression, EphA4 null astrocytes are less responsive to stimuli that require cytoskeletal regulation [31]. In addition, lack of EphA4 on astrocytes would affect interactions with surrounding ephrin-expressing cells, including oligodendrocytes/myelin, that express ephrin-B3 [32] and which would normally also interact with EphA4-expressing axons. Blocking of EphA4 using a decoy receptor produced a modest delay in disease severity reflective of the observations in the EphA4 knockout mice. This raises the potential for treatment of human MS, possibly in combination with current immunomodulatory therapy.

## Supporting Information

Figure S1 **Fluoromyelin staining of EAE-affected spinal cords.** Representative sections from WT and EphA4 KO mice with grade 2.5 EAE, stained for myelin using Fluoromyelin stain. These were counterstained with the nuclear stain DAPI to identify regions containing lesion sites. Only areas containing inflammatory infiltrates showed gross loss of myelin staining. Scale bar 100 µm.
(TIF)
