# Supplementary figures and images for: EphA4 Receptor Tyrosine Kinase Is a Modulator of Onset and Disease Severity of Experimental Autoimmune Encephalomyelitis (EAE)
Source: PLoS One. 2013 Feb 4;8(2):e55948. doi: 10.1371/journal.pone.0055948 (PMC3563632; doi:10.1371/journal.pone.0055948)

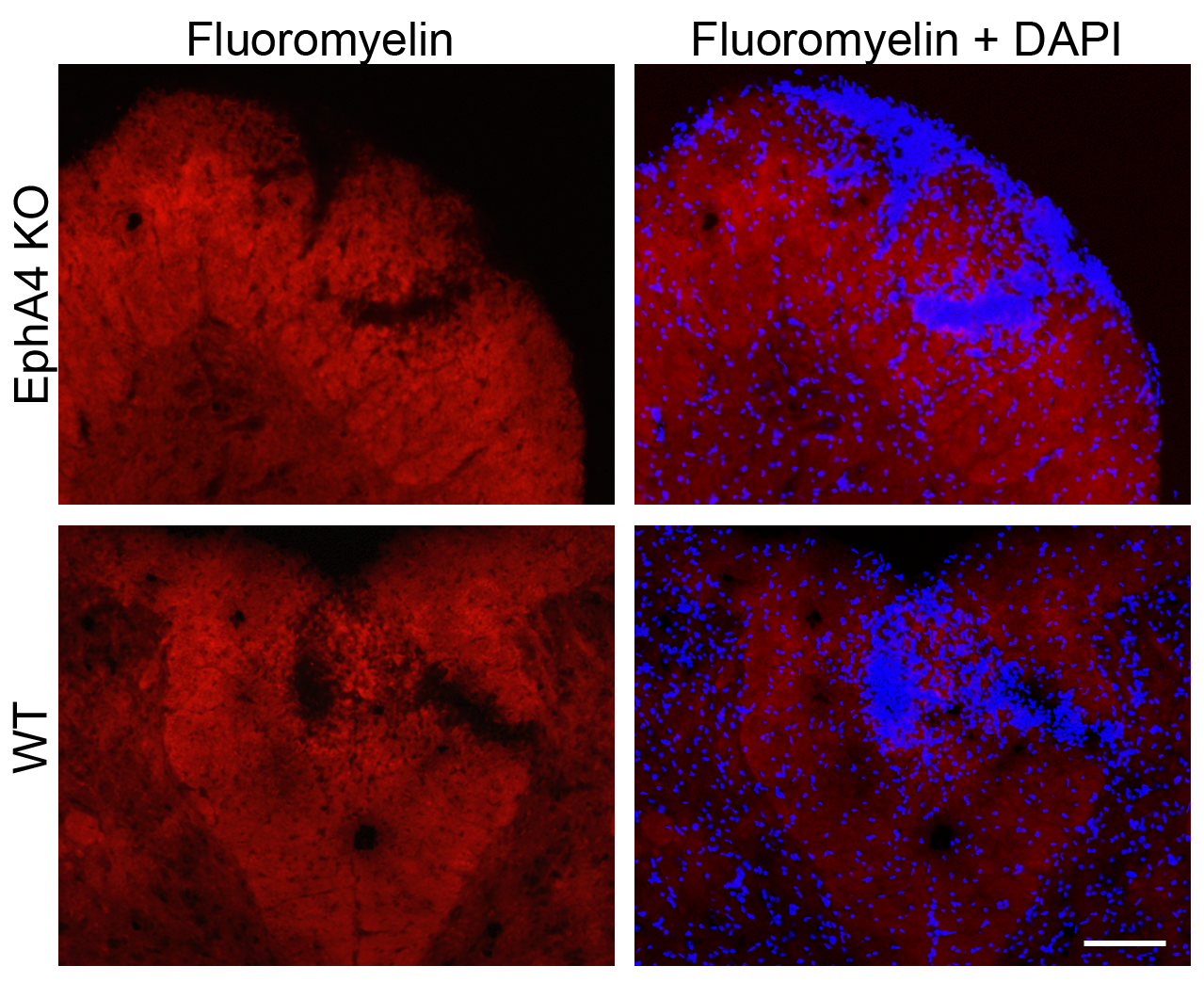

Supplement: Figure S1 — Fluoromyelin staining of EAE-affected spinal cords. Representative sections from WT and EphA4 KO mice with grade 2.5 EAE, stained for myelin using Fluoromyelin stain. These were counterstained with the nuclear stain DAPI to identify regions containing lesion sites. Only areas containing inflammatory infiltrates showed gross loss of myelin staining. Scale bar 100 µm. (TIF) [file pone.0055948.s001.tif]
